# Long-Term Survival after Coronary Artery Surgical Revascularization—Does Ambient Temperature Matter?

**DOI:** 10.3390/medicina60081220

**Published:** 2024-07-27

**Authors:** Tomasz Urbanowicz, Krzysztof Skotak, Jakub Bratkowski, Anna Olasińska-Wiśniewska, Krzysztof J. Filipiak, Michał Michalak, Kajetan Grodecki, Krystian Szczepański, Andrzej Tykarski, Beata Krasińska, Zbigniew Krasiński, Aleksandra Krasińska-Płachta, Marek Jemielity

**Affiliations:** 1 Cardiac Surgery and Transplantology Department, Poznan University of Medical Sciences, 61-701 Poznan, Poland; 2 Institute of Environmental Protection–National Research Institute, 02-170 Warsaw, Poland; 3 Institute of Clinical Science, Maria Sklodowska-Curie Medical Academy, 00-136 Warsaw, Poland; 4 Department of Computer Science and Statistics, Poznan University of Medical Sciences, 60-806 Poznan, Poland; michal@ump.edu.pl; 5 1st Cardiology Department, Warsaw University of Medical Sciences, 02-091 Warsaw, Poland; 6 Department of Hypertensiology, Angiology and Internal Medicine, Poznan University of Medical Sciences, 61-701 Poznan, Poland; 7 Department of Vascular and Endovascular Surgery, Angiology and Phlebology, Poznan University of Medical Sciences, 61-701 Poznan, Poland; 8 Department of Ophthalmology, Poznan University of Medical Sciences, 61-107 Poznan, Poland; alex.krasinska@gmail.com

**Keywords:** tropical nights, ambient temperature dyslipidemia, CAD, OPCAB

## Abstract

*Background and Objectives:* The progression of global warming results in an increased exposure to extreme heat, leading to exaggeration of preexisting diseases and premature deaths. The aim of the study was to present possible risk factors for all-cause long-term mortality in patients who underwent surgical revascularization, including an assessment of the influence of ambient temperature exposure. *Materials and Methods:* Retrospective analysis included 153 (123 (80%) males and 30 (20%) females) patients who underwent off-pump revascularization and were followed for a median time of 2533 (1035–3250) days. The demographical, clinical data and ambient temperature exposure were taken into analysis for prediction of all-cause mortality. Individual exposure was calculated based on the place of habitation. *Results:* In the multivariate logistic regression model with backward stepwise elimination method, risk factors such as dyslipidaemia (*p* = 0.001), kidney disease (*p* = 0.005), age (*p* = 0.006), and body mass index (*p* = 0.007) were found to be significant for late mortality prediction. In addition to traditional factors, environmental characteristics, including tropical nights (*p* = 0.043), were revealed to be significant. *Conclusions:* High night-time ambient temperatures known as tropical nights may be regarded as additional long-term mortality risk factor after surgical revascularization.

## 1. Introduction

The progression of global warming has resulted in an increased exposure to extreme heat, leading to the deterioration of preexisting diseases and premature deaths [1]. In epidemiological studies, many investigators have considered daytime maximal temperatures and daily mean temperatures indexes as potential morbidity risk factors [2]. High temperatures may influence fluid body balance, blood homeostasis, and viscosity, and may alter human behavior, the transmission of disease, and air quality. Moreover, the fall in temperature overnights is an important issue impacting human recovery after daytime heat [3]. It was emphasized that if the overnight temperature does not fall optimally, then organisms are unable to properly cool down and acquire healing-related complications. This issue is particularly important in particular subpopulations, such as elderly subjects, who are burdened with cardiovascular, cerebrovascular or kidney diseases. Roye in his analysis presented the association between hot nights and premature mortality [4]. Importantly, a relationship between exposure variables and mortality persists significantly up to a lag of 1–2 days. Thus, the effect of extreme temperatures is broader and more complex. Murage et al. revealed an increased risk of stroke and overall mortality related to warm nights preceded by hot days [5].

Coronary artery disease remains one of the main epidemiological challenges faced by the current population. Satisfactory results of interventional therapy in multivessel coronary disease are related to percutaneous and surgical revascularization [6]. In the aging population, characterized by increased life expectancy, the proper identification of mortality risk factors is of utmost importance. Environmental and genetic factors, along with lifestyle, are posed as risk influences for coronary disease in addition to co-morbidities such as diabetes mellitus, hypertension, and hyperlipidemia [7]. The increased risk of acute coronary syndromes [8] and chronic coronary disease progression [9,10] related to environmental factors has been already postulated. Patients treated surgically due to coronary artery disease are recommended to adhere to medication use, obtain a strict lipid profile and body weight, and to stop smoking. However, environmental and temperature instructions are not routinely stated.

The aim of the study was to present the significance of non-classical possible risk factors of all-cause long-term mortality in patients who underwent surgical revascularization.

## 2. Materials and Methods

### 2.1. Patients

Retrospective analysis included 153 patients (123 (80%) males and 30 (20%) females, median (Q1–3) age of 66 (60–70) years) operated on in the Cardiac Surgery Department using off-pump revascularization technique and followed for a median time of 2533 (1035–3250) days. The study group comprised 103 (67%) patients with multivessel coronary artery disease and 50 (33%) with left main coronary disease from the western and central parts of Poland. None of the patients were professionally active and all were pensioners. Regarding the indoor characteristics, none of the participants had air-conditioning.

Demographic and clinical data were collected at admission. All procedures were performed through median sternotomy using off-pump technique (OPCAB, off-pump coronary artery bypass grafting). Procedural data were assessed. Post-discharge outcomes, with survival rate, were followed-up. The exposure to meteorological factors in the observation period was provided by the Institute of Environmental Protection and analysed together with demographic and clinical data.

Co-morbidities included arterial hypertension (*n* = 102, 67%), diabetes mellitus (*n* = 43, 28%), dyslipidaemia (*n* = 47, 31%), peripheral artery disease (*n* = 20, 13%), and chronic kidney disease (*n* = 5, 3%). The median body mass index (BMI) value was 28.7 (25.9–31.2). Thirty-seven (24%) patients reported chronic tobacco use.

### 2.2. Environmental Factors (Ambient Temperature Exposure)

Ambient temperature exposure was calculated individually for each patient and presented as median daily minimal and maximal values followed by days with median temperatures exceeding 25 and 30 degrees Celsius. Tropical nights were estimated as number of days with a minimal temperature above 20 degrees Celsius and were calculated for each patient individually. Annual exposure to the number of days with ambient with temperature crossing 0 °C was individually estimated. Cold waves were defined as 3 consecutive days with minimum daily ambient temperatures less than −20 °C.

Estimation exposures of climate temperature parameters (inc. mean daily winter temperatures and number of tropical nights—days with minimum temperature > 20 °C) based on data from international climate downscaling initiative EURO-CORDEX models [11] provided high-resolution climate projections of 12.5 km for Europe [12,13]. For the final analysis, we used Representative Concentrations Pathways emission scenarios—RCP4.5 [14], described by The Intergovernmental Panel on Climate Change (IPCC) in the Fifth Assessment Report (AR5) [14].

In our simulation, ERA-Interim re-analysis [15] and daily temperature from the European Climate Assessment & Dataset (ECA&D) [16] were used for boundary conditions. Observations from the Polish Institute of Meteorology and Water Management were assimilated for data calibration [17].

The high-resolution temperature-calculated data used in this study are widely available from the Institute of Environmental Protection—National Research Institute in Poland (IEP-NRI) web page [18].

### 2.3. Statistical Analysis

The normality of the distribution of variables was tested with the Shapiro–Wilk test. The *t*-test, Cochran–Cox test, Mann–Whitney test, and Fisher’s exact test were used where applicable to compare the variables between the two groups. Logistic regression was performed to analyze the predictors of long-term mortality. Receiver operator characteristic (ROC) analysis was carried out. Spearman correlation analysis was used to describe the correlation between the variables. Statistical analysis was performed using Statistica 13 by TIBCO. *p* < 0.05 was considered statistically significant.

### 2.4. Bioethics Committee Approval

Informed consent was obtained from all participants. The study was conducted in accordance with the Declaration of Helsinki and approved by the Institutional Review Board (or Ethics Committee) of Poznan University of Medical Sciences, Poznan, Poland (protocol code 55/20 from 16 January 2020), for studies involving humans.

## 3. Results

There were 32 (21%) deaths in the presented group within 2533 (1035–3250) days of follow-up. There were no perioperative deaths. The median number of performed anastomoses was 2.0 (2.0–3.0). Complete arterial revascularization was performed in 33 (22%) patients. The median hospitalization time was 10 (8–12) days. Following the surgery, all patients were treated according to current guidelines regarding coronary artery disease, including B-blockers, antiplatelets, statins, and angiotensin-converting enzyme inhibitor/angiotensin II receptor blockers.

There were no significant differences in the demographical and clinical profile between survivors and deceased groups, as presented in Table 1.

### 3.1. Temperature Characteristics

Except for traditional risk factors, environmental factors were analyzed, including temperature exposure in the place of habitation for each patient. Daily, seasonal, and yearly temperature characteristics were estimated. The exposure to heat events named tropical nights was calculated among ambient temperature characteristics. They were defined as a number of days with minimal temperature above 20 degrees Celsius, reaching the median values of 20.5 (10.1–31.0) days. There were no significant differences in exposure to ambient temperature characteristics between subgroups, as presented in Table 2.

### 3.2. Logistic Regression Analysis

Uni- and multivariable analysis for predicting late mortality after coronary artery bypass grafting, including environmental factors, was performed and presented in Table 3.

The univariable analysis presented the significant value of age (*p* = 0.001) and kidney disease (*p* = 0.046) for the prediction of all-cause mortality after the OPCAB procedure. In the multivariate logistic regression model with a backward stepwise elimination method (Table 3), traditional risk factors such as dyslipidemia (*p* = 0.001), kidney disease (*p* = 0.005), age (*p* = 0.006), and BMI (*p* = 0.007) were found to be significant for late mortality prediction. In addition to the factors mentioned above, environmental characteristics such as tropical nights (*p* = 0.043) were revealed to be significant.

**Figure 1:**
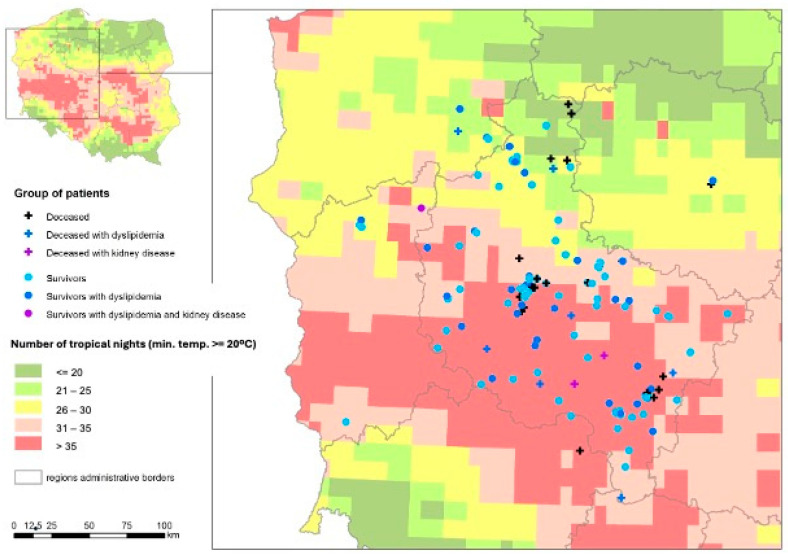
Tropical night exposure in 2011–2023 in presented groups related to geographic distribution.

The exposure to sum of tropical nights in the 2011–2023 period related to geographic characteristics in the western and central parts of Poland in deceased and survived patients is presented in Figure 1.

## 4. Discussion

Our analysis points out the significance of environmental and traditional mortality risk factors after surgical revascularization. We focused on the prediction of late all-cause mortality following the off-pump procedure. The regression model indicated the influence of kidney disease and dyslipidemia. Moreover, the analysis showed the clinical relevance of tropical night exposure on late mortality.

In the previous epidemiological analysis by Alahmad et al. [19], there was an association between increased mortality related to cardiovascular diseases and exposure to extreme—either hot or cold—ambient temperatures. We showed that a combination of traditional, demographical, and clinical risk factors and environmental features may enhance the prediction of long-term outcomes. Moreover, our analysis may help to explain differences in survival of patients presenting seemingly similar cardiovascular profiles. The difference in the exposure to ambient temperatures, including tropical nights, may change the body’s response to therapy and increase mortality.

Clinical features which were shown as significant in our logistic regression analysis are already known in the cardiovascular high-risk profiles. The dyslipidemia is an established risk factor of coronary artery disease progression [20]. Most publications reporting the significance of cardiovascular disease progression related to lipid hemostasis disturbances are accompanied by interference between dyslipidemia and ambient temperature. As presented in the Pirillo et al. review [21], in the last 30 years, the global burden of dyslipidemias has been observed and is reported to increase the risk of cardiovascular diseases. Therapies lowering the low-density lipoprotein cholesterol (LDL) are the cornerstone for reducing cardiovascular complications [21]. Therefore, current guidelines recommend aggressive lipid-lowering therapeutic options to decrease cardiovascular risk [20]. The relation between ambient temperature and high-density lipoprotein cholesterol (HDL) and levels was presented by Halonen et al. [22]. In their analysis, the HDL decrease was inversely correlated with ambient temperature contrary to the linear relation with the LDL levels. Xu et al. [23] presented the significant effect of heat on hospitalizations for stroke treatment in individuals with dyslipidemia, depending on gender.

Moreover, our study indicated a strong relationship between kidney disease and long-term outcomes in patients with complex coronary disease treated by surgical revascularization. The interplay between chronic kidney disease and the cardiovascular system, resulting in increased morbidity and mortality risk, was pointed out in previous publications [24]. Kidney dysfunction-related increased risk of cardiovascular events and all-cause mortality is associated with the cardiovascular-kidney-metabolic axis [25]. Novel drugs such as glucagon-like peptide-1 receptor agonists, regardless of structural homology, reduced the risk of individual major adverse cardiovascular events (MACE), all-cause mortality, and hospital admissions [26]. The possible role of heat on cardiovascular events has gained significant attention in recent publications. Liu et al. [27] postulated the relation between dehydration and a systemic inflammatory response, enhanced renin-angiotensin system activation, and ambient temperature-related sympathetic reactivity. Ichicki et al. [28] pointed out the inverse relation between glomerular filtration rate (GFR) and ambient temperature. Warm, ambient temperatures influence obesity-related outcomes [29].

In our analysis, obesity was also identified as a mortality risk factor consistent with previous studies that indicated the association between BMI and overall mortality and specific causes of death such as mental, behavioral, and neurological modes [30].

Orkaby et al., in their analysis, showed a significant reduction in cardiovascular mortality in elderly patients associated with optimal lipid-lowering therapies [31]. The aging population presents a higher mortality risk related to multiple co-morbidities and their advancement [32]. Our analysis confirmed age as one of the possible risk factors of late mortality, which could be related to the increased prevalence of co-morbidities.

The main novelty of our analysis is considered in terms of environmental influence on patients with cardiovascular profiles who underwent surgical coronary revascularisation. We aimed to point out the significance of environmental factors for long-term survival. The adverse effects of environmental characteristics have already been proven, including our previous analysis reporting the progression of atherosclerotic lesions related to exposure to air pollution [10,33]. Tropical nights, defined by the number of days with minimal temperature above 20 degrees Celsius, should draw clinical attention to the influence of ambient temperature on human health. Temperature drops during the nighttime are detrimental. Observational studies have pointed out the relationship between heat waves and increased mortality risk [34]. Our analysis indicates the importance of nighttime temperature; similarly, He et al. [1] revealed the growing role of nighttime warming in heat-related health effects. Several authors underlined the association between hot nights and increased mortality [1,35,36]. Saucy et al. [37] reported that heat-related mortality was driven mainly by ischaemic heart disease, myocardial infarction, and arterial hypertension.

The composition of traditional and environmental predictors revealed in our logistic analysis is not causal. Heat exposure causes general vasodilatation, which is necessary for body temperature homeostasis, but leads to increased heart rate and organ oxygen demand [37,38]. Dehydration is an important issue and may be exaggerated because water consumption is decreased during the nighttime compared to the daytime. This problem is particularly strongly associated with kidney disease, which may be worsened by dehydration and hypovolemia, leading to even a prerenal form of acute kidney injury, and age, since older adults tend to intake less fluids, have changed thirst perception, worse adaptation to temperature changes, and avoid air-conditioning [1,39]. Moreover, multiple co-morbidity, especially cardiovascular diseases, coronary artery disease, heart failure, and arterial hypertension, are often associated with pharmacotherapy, including diuretics, which may deteriorate hypovolemia. Challenges in the control of arterial hypertension depending on temperature changes have been described [39]. It should be underlined that patients with cardiovascular issues tolerate ambient temperatures worse than healthy subjects in whom thermoregulation, though loaded with ambient temperatures, is better. Based on the current report, we suggest that patients treated surgically due to coronary artery disease should be recommended to protect themselves from extreme temperatures, particularly at nighttime, to avoid increased risk of morbidity and mortality.

### Study Limitation

The study was based on a limited number of patients as personalised exposure to ambient temperature was calculated individually for each patient and related to their place of habitation. High values of confidence interval results can be explained by this reason. The personalised approach, despite a limited number of patients, allowed for exact analysis of ambient temperature exposure. In our analysis, we focused on ambient temperature characteristics excluding the interplay between coronary artery disease progression and air pollution [33], especially related to city aggromelations [10] that were presented in previous analyses.

## 5. Conclusions

High nighttime ambient temperatures known as tropical nights can be regarded as additional predictor of long-term mortality after surgical revascularization in addition to traditionally established demographical and clinical risk factors.

## Figures and Tables

**Table 1:** The demographical and clinical characteristics of survivors and deceased group.

Parameters	Survivors *n* = 121	Deceased *n* = 32	*p*
Demographical:			
Age (years, median (Q1–Q3)	65 (59–70)	67 (64–74)	0.057
Sex (Male (%)/Female (%))	100 (83)/21 (17)	23 (72)/9 (28)	0.175
BMI (median (Q1–Q3))	28.3 (25.8–30.6)	29.4 (27.4–31.9)	0.066
Clinical:			
Arterial Hypertension (*n*, (%))	84 (69)	18 (56)	0.162
Diabetes mellitus (*n*, (%))	36 (30)	7 (22)	0.381
Dyslipidaemia (*n*, (%))	39 (32)	8 (25)	0.434
Peripheral artery disease (*n*, (%))	17 (14)	3 (9)	0.489
Kidney dysfunction (*n*, (%))	3 (3)	2 (6)	0.291
Tobacco use (*n*, (%))	29 (24)	8 (25)	0.906
Coronary disease:			
Left main disease (*n*, (%))	42 (35)	8 (25)	0.301
3 vessel disease (*n*, (%))	39 (32)	11 (34)	0.834
2 vessel disease (*n*, (%))	41 (34)	13 (41)	0.535
Surgical procedure:			
Number of grafts (*n*, median (Q1–Q3)	2 (2–3)	2 (2–2.3)	0.189
Arterial revascularization (*n*, median (Q1–Q3)	30 (25)	3 (9)	0.061
Observation days (median (Q1–Q3)	2017 (974–3250)	2872 (1650–3250)	0.355

Abbreviations: BMI—body mass index, *n*—number.

**Table 2:** Environmental exposure in survivors and deceased groups.

Parameters	Survivors *n* = 121	Deceased *n* = 32	*p*
Daily temperature extremities:			
below 0 °C (number of days/year)	24.3 (22.5–25.9)	24.8 (22.7–26.4)	0.321
below −10 °C (number of days/year)	10.6 (10.2–11.1)	10.5 (10.2–11.2)	0.605
above 25 °C (number of days/year)	44.0 (40.8–45.6)	44.0 (40.8–45.3)	0.613
above 30 °C (number of days/year)	10.6 (9.4–10.9)	10.6 (9.4–10.8)	0.569
Seasonal characteristics			
Spring min (mean temperature throughout the season)	4.4 (4.1–4.5)	4.4 (4.1–4.5)	0.647
Spring mean (mean temperature throughout the season)	8.9 (8.6–8.9)	8.9 (8.6–8.9)	0.533
Spring max (mean temperature throughout the season)	3.1 (2.8–3.3)	3.1 (2.8–3.3)	0.597
Summer min (mean temperature throughout the season)	6.3 (6.1–6.4)	6.3 (6.1–6.4)	0.439
Summer mean (mean temperature throughout the season)	18.7 (18.4–18.8)	18.7 (18.4–18.8)	0.660
Summer max (mean temperature throughout the season)	24 (23.7–24.0)	23.9 (23.7–24.0)	0.597
Autumn min (mean temperature throughout the season)	6.3 (6.1–6.4)	6.3 (6.1–6.4)	0.439
Autumn mean (mean temperature throughout the season)	10.2 (9.9–10.2)	10.1 (9.9–10.2)	0.359
Autumn max (mean temperature throughout the season)	14.1 (13.8–14.2)	14.1 (13.8–14.2)	0.548
Winter min (mean temperature throughout the season)	0.7 (0.5–1.0)	0.7 (0.5–1.0)	0.438
Winter mean (mean temperature throughout the season)	3.1 (2.8–3.3)	3.1 (2.8–3.3)	0.430
Winter max (mean temperature throughout the season)	−2.1 (−2.3–−1.9)	−2.1 (−2.3–−2.1)	0.686
Yearly characteristics:			
mean (°C)	9.6 (9.4–9.7)	9.6 (9.4–9.7)	0.708
max (°C)	5.5 (5.3–5.6)	5.5 (5.3–5.6)	0.599
min (°C)	13.8 (13.4–13.8)	13.7 (13.4–13.8)	0.376
Tropical nights (number of nights/year)	3.6 (3.1–3.7)	3.6 (3.1–3.7)	0.617
Tropical night exposure (days)	19.8 (10.0–30.9)	22.9 (13.0–31.2)	0.444
Cold wave (days/year)	5.4 (5.4–5.6)	5.5 (5.3–5.7)	0.837
Number of days with temperature crossing 0 °C (days/year)	57.3 (56.2–59.4)	58 (56.2–59.4)	0.925

Abbreviations: C—Celsius, *n*—number.

**Table 3:** Uni- and multivariable regression analysis for long-term mortality prediction.

	Univariable Analysis			Multivariable Analysis		
	OR	95% CI	*p*	OR	95% CI	*p*
Demographical:						
Sex	0.48	0.22–1.05	0.065	-	-	-
Age	1.08	1.03–1.13	0.001 *	1.13	1.04–1.23	0.006
BMI	1.08	0.99–1.17	0.092	1.22	1.06–1.41	0.007
Clinical:						
HA	1.60	0.72–3.56	0.249	-	-	-
Dyslipidaemia	1.68	0.73–3.92	0.227	18.8	3.43–102.4	0.001
DM	1.40	0.59–3.31	0.440	-	-	-
Peripheral artery disease	1.14	0.34–3.80	0.832	-	-	-
Kidney disease	4.38	1.03–18.66	0.046 *	39.61	2.98–527.4	0.005
Tobacco use	1.95	0.84–4.51	0.118	-	-	-
Number of grafts	0.56	0.34–0.93	0.026	-	-	-
Arterial revascularization	0.69	0.20–2.32	0.548	-	-	-
Perioperative complications	1.91	0.57–6.34	0.292	-	-	-
Mild LVEF dysfunction	1.09	0.43–2.74	0.086	-	-	-
Ambient:						
Days < −10 C	1.02	0.65–1.61	0.923	-	-	-
Days > 25 C	1.01	0.93–1.10	0.866	-	-	-
Tropical nights	1.10	0.58–2.08	0.768	10.3	1.08–98.5	0.043
Seasonal mean spring	1.06	0.26–4.22	0.938	-	-	-
Seasonal mean summer	1.04	0.38–2.82	0.948	-	-	-
Seasonal mean autumn	1.00	0.29–4.32	1.000	-	-	-
Seasonal mean winter	0.89	0.28–2.85	0.847	-	-	-
Cold waves	1.31	0.24–7.13	0.756	-	-	-
Cross 0 °C	1.03	0.88–1.21	0.715	-	-	-

Abbreviations: BMI—body mass index, CI—confidence interval, Cross—number of days with temperature crossing 0 °C (days/year). DM—diabetes mellitus, HA—arterial hypertension, LVEF—left ventricular ejection fraction, OR—odd ratio. * statistically significant.

## Data Availability

The data will be available for three years following the publication upon reasonable request by contacting the corresponding author via e-mail.
